# Obesity and Male Reproduction: Do Sirtuins Play a Role?

**DOI:** 10.3390/ijms23020973

**Published:** 2022-01-16

**Authors:** Federica Barbagallo, Sandro La Vignera, Rossella Cannarella, Laura M. Mongioì, Vincenzo Garofalo, Claudia Leanza, Marta Marino, Aldo E. Calogero, Rosita A. Condorelli

**Affiliations:** Department of Clinical and Experimental Medicine, University of Catania, 95123 Catania, Italy; federica.barbagallo11@gmail.com (F.B.); sandrolavignera@unict.it (S.L.V.); rossella.cannarella@phd.unict.it (R.C.); lauramongioi@hotmail.it (L.M.M.); vgarofalo985@gmail.com (V.G.); claudia.leanza.95@gmail.com (C.L.); martamarino@outlook.com (M.M.); acaloger@unict.it (A.E.C.)

**Keywords:** sirtuins, obesity, male fertility, sperm parameters, resveratrol

## Abstract

Obesity is a major current public health problem of global significance. A progressive sperm quality decline, and a decline in male fertility, have been reported in recent decades. Several studies have reported a strict relationship between obesity and male reproductive dysfunction. Among the many mechanisms by which obesity impairs male gonadal function, sirtuins (SIRTs) have an emerging role. SIRTs are highly conserved nicotinamide adenine dinucleotide (NAD+)-dependent deacetylases that play a role in gene regulation, metabolism, aging, and cancer. SIRTs regulate the energy balance, the lipid balance, glucose metabolism, and adipogenesis, but current evidence also indicates a role for SIRTs in male reproduction. However, the majority of the studies have been conducted in animal models and very few have been conducted with humans. This review shows that SIRTs play an important role among the molecular mechanisms by which obesity interferes with male fertility. This highlights the need to deepen this relationship. It will be of particular interest to evaluate whether synthetic and/or natural compounds capable of modifying the activity of SIRTs may also be useful for the treatment of obesity and its effects on gonadal function. Although few studies have explored the role of SIRT activators in obesity-induced male infertility, some molecules, such as resveratrol, appear to be effective in modulating SIRT activity, as well as counteracting the negative effects of obesity on male fertility. The search for strategies to improve male reproductive function in overweight/obese patients is a challenge and understanding the role of SIRTs and their activators may open new interesting scenarios in the coming years.

## 1. Introduction

### 1.1. Obesity: A Global Health Problem

Obesity is a major public health problem of global significance. According to the World Health Organization (WHO), worldwide obesity has nearly tripled since 1975. Approximately 1.9 billion adults were overweight in 2016 and approximately 650 million were obese [1]. Obesity is linked to more deaths than being underweight [1].

Obesity and being overweight are defined as an abnormal or excessive fat accumulation that may impair health. The WHO defines being overweight as having a body mass index (BMI) greater than, or equal to, 25 kg/m^2^; obesity is defined as a BMI greater than 30 kg/m^2^; and severe obesity is defined as a BMI greater than 40 kg/m^2^ [1].

An increased BMI is associated with an elevated premature mortality risk [2], as well as several comorbidities including cardiovascular diseases, diabetes mellitus (DM), musculoskeletal disorders, sleep apnea syndrome, nonalcoholic fatty liver disease (NAFLD), and some cancers [3]. Consequently, research into obesity and obesity-related diseases has become a major need today.

### 1.2. Male Infertility: A Problem of Epidemic Dimensions

The WHO defines infertility as the failure to achieve a clinical pregnancy after 12 months or more of regular unprotected sexual intercourse [4]. In the third decade of the new millennium, infertility represents a highly prevalent global condition, affecting 15% of all couples of reproductive age in industrialized countries [5]. The inability to conceive affects couples worldwide and causes emotional and psychological distress in both men and women.

Infertility has been considered only a woman’s problem for a long time. During the last 20 years, the attention of the scientific world to male-related fertility issues has grown exponentially. Agarwal and colleagues showed that at least 30 million men worldwide were infertile [5]. Half of the cases of couple infertility recognized a male factor of infertility [6].

The prevalence of infertility has increased notably in recent decades. A progressive decline in sperm quality has been described [7,8]. Lifestyle and environmental factors, such as smoking [9], obesity [10], endocrine disruptors [11,12], an exposure to heavy metals [13,14], or psychological stress [15] may have a major role in the decreased sperm quality.

The growing interest in this field shows that lifestyle factors play a key role in reproductive health and may influence fertility.

### 1.3. The Relationship between Obesity and Male Reproduction

Among the lifestyle factors, nutrition has a profound influence on fertility. Several studies have shown that obesity has a negative impact on male fertility [16]. A meta-analysis reported that couples with an obese male partner have a significantly higher risk of infertility than couples with normal-weight male partners (OR = 1.66, 95% CI 1.53–1.79). Furthermore, male obesity negatively affects the success of assisted reproductive technology (ART) [17]. An increased BMI in men is associated with a significant reduction in pregnancy (OR 0.78, 95% CI 0.63 to 0.98, *p* = 0.03) and live birth (OR 0.88, 95% CI 0.82 to 0.95, *p* = 0.001) rates in intracytoplasmic sperm injection (ICSI) cycles [18].

Obesity can impair the male reproductive system by its effects on erectile function and impairing semen quality. Several studies demonstrated that BMI correlates negatively with the sperm concentration [19,20], motility [21], and normal morphology [22]. A systematic review and meta-analysis showed that obesity and being overweight is associated with a higher prevalence of azoospermia and oligozoospermia [23]. Obesity is also associated with an increased sperm DNA fragmentation rate [24,25]. Regarding other biofunctional sperm parameters, obesity is associated also with abnormal sperm chromatin compactness, a high percentage of spermatozoa with a low mitochondrial membrane potential (MMP), and phosphatidylserine (PS) externalization, an early marker of apoptosis [22].

Several mechanisms have been hypothesized to explain obesity-induced sperm damage, including abnormal reproductive hormone levels, insulin resistance, the altered production of adipokines, a higher scrotal temperature, increased oxidative stress, and chronic systemic inflammation [10]. The excessive visceral fat leads to a reduction in the sex hormone-binding globulin (SHBG), the total and free testosterone (T), inhibin B, and an increased conversion of testosterone to estradiol [E_2_] due to a greater aromatase activity [25,26]. The reduction in T levels negatively impacts spermatogenesis since proper concentrations of intratubular T are essential for the adhesion of Sertoli cells to the developing germ cells [27]. Obesity also promotes epigenetic modifications that can be transmitted to offspring [28].

Furthermore, excessive visceral fat produces pro-inflammatory cytokines, such as interleukin-6 (IL-6) and tumor necrosis factor α (TNFα), which cause a low-grade systemic inflammation [29]. In obese patients, an imbalance between oxidative and antioxidant systems results in an increased production of reactive oxygen species (ROS). At physiological levels, ROS are essential for the physiological processes of spermatogenesis. Conversely, at high concentrations, they can oxidize and damage DNA, proteins, and lipids [30].

Obesity also causes insulin resistance and, in turn, hyperinsulinemia that lowers SHBG production by the liver, leading to higher levels of E_2_. The excess of E_2_ inhibits the hypothalamic-pituitary-gonadal axis and this results in a reduction in T production [10]. The production of adipokines is also altered in obese patients. A higher secretion of leptin by the adipose tissue, and consequent leptin resistance, impairs male fertility at both central and peripheral levels. The excess of leptin decreases gonadotropin-releasing hormone (GnRH) secretion, mainly suppressing the kisspeptin neuronal function which can directly impair spermatogenesis [10]. Therefore, an insulin excess and dysregulated adipokines have a detrimental effect on testicular function, giving rise to a “metabolic” form of male hypogonadism. However, a complex, bi-directional crosslink between visceral adipose tissue dysfunction, systemic insulin resistance, and testicular malfunctioning has been demonstrated. Indeed, low testosterone levels further deteriorate insulin sensitivity and promote adipocyte proliferation and an increase in body fat, creating a repeating cycle [31].

Among the complex molecular mechanisms that are behind obesity-induced male infertility, new molecules are emerging on the horizon, such as sirtuins (SIRTs). Recent literature suggests that the role of SIRTs on obesity and male fertility operates through different mechanisms. Therefore, this review aims to investigate the role of SIRTs in obesity-induced male infertility.

## 2. Sirtuins, Obesity and Male Fertility: Is There a Relationship?

### 2.1. What Are Sirtuins?

SIRTs are highly conserved nicotinamide adenine dinucleotide (NAD+)-dependent deacetylases that play a role in gene regulation, metabolism, aging, and cancer [10]. SIRTs are found throughout the realms of life and are phylogenetically conserved from archeobacteria to humans [32].

The function of the SIRT family is mostly related to the change in the state of protein acylation. They catalyze the deacetylation of proteins by breaking the bonds between NAD+ and niacinamide ribosomes, transferring the acetylated groups from proteins to adenosine-5′-diphosphate(ADP)-ribose, then releasing the deacetylated products [33].

In mammals, seven types of SIRTs (SIRTs 1-7) have been identified with different structures, cellular localizations, and tissue expressions. Eukaryotic SIRTs have been divided into four broad phylogenetic groups based on sequence similarities: class I (SIRT1, SIRT2, and SIRT3), class II (SIRT4), class III (SIRT5), and class IV (SIRT6 and SIRT7). There is no obvious correlation between this classification and the specific biological functions of SIRTs. Based on their intracellular localization, SIRTs are divided into nuclear, cytoplasmic, and mitochondrial SIRTs [34]. SIRT1, SIRT6, and SIRT7 are localized in the nucleus, but some cells have a cytosolic expression of SIRT1. They participate in the regulation of energy metabolism, stress, inflammatory responses, DNA repair (SIRT1 and SIRT6), and rDNA transcription (SIRT7) via modifications of transcription factors, cofactors, and histones. SIRT2 is localized in the cytoplasm and primarily plays a role in cell cycle control. SIRT3, SIRT4, and SIRT5 are mostly found in mitochondria. SIRT3 participates in the regulation of metabolic enzymes involved in glycolysis, fatty acid oxidation, ketone body synthesis, and amino acid catabolism; apoptosis; and oxidative stress pathways, and may also regulate cellular metabolism both at the transcriptional and post-transcriptional levels. SIRT4 works as an ADP-ribosylase. Lastly, SIRT5 has potent demalonylation and desuccinylation enzymatic activities and is involved in the regulation of amino acid catabolism. Despite variable lengths and sequences, all SIRTs have a highly conserved catalytic core region consisting of approximately 275 amino acids, forming a Rossmann-fold domain, which is characteristic of NAD+/NADH binding proteins, and a zinc-binding domain, connected by several loops. Outside the catalytic core, SIRT enzymes have variable N- and C-terminal regions that drive their enzymatic activities, substrate bindings, and subcellular localizations [35].

SIRTs are present in different tissues. These include the hypothalamus, liver, brain, heart, kidney, pancreatic islets, skeletal muscles, adipocytes, testicular tissue, and oocytes. Therefore, they play an important role in the regulation of cellular homeostasis and, in particular, metabolism, inflammation, oxidative stress, and senescence [36] by activating several pathways and enzymes. These then comprehend the peroxisome proliferator-activated receptor-gamma coactivator (PGC)-1α, the peroxisome proliferator-activated receptor gamma (PPAR-γ), the adenosine monophosphate-activated protein kinase (AMPK), the Forkhead box transcription factor O1 (FOXO1), and the liver X receptor (LXR) [10].

Some SIRTs are also involved in chromatin structure remodeling and accessibility. Through the modification of histones, as well as transcription factors and co-regulators, SIRTs regulate the expression of various genes and, particularly, those involved in the stress response. The SIRT expressions and activities of SIRT enzymes are highly sensitive to several environmental factors, including calorie restrictions (CR), exercise, and cold exposure [35]. When calories are restricted, the flux of carbons through mitochondrial oxidation shifts the NAD+/NADH balance towards the oxidized state, thus establishing a redox environment that promotes the activity of SIRTs [37].

### 2.2. Sirtuins and Obesity

Many studies in recent decades have shown the role of SIRTs in the metabolism, secretion, and sensitivity of insulin, as well as the metabolic homeostasis of the body [38,39]. SIRTs regulate insulin secretion, sensitivity, and mobilization or oxidation of stored fat by interacting with several transcription factors and adipokines [40].

The roles of SIRTs in inflammation and the antioxidant balance significantly contribute to the onset of insulin resistance, obesity, and other metabolic diseases. SIRT1 seems to contribute to the maintenance of a balance between oxidative and antioxidant systems, protecting cells from oxidative stress damage. Consequently, low levels of SIRT1 and SIRT3 lead to the defective function of the electron transport chain, increasing the production of ROS [41]. The absence of SIRTs, or their low levels, are linked to the pathogenesis of insulin resistance, obesity, and DM [42,43]. SIRT1 positively regulates insulin secretion and protects pancreatic ß-cells from oxidative stress and inflammation. SIRTs protect against the onset of DM by several mechanisms, including the modulation of insulin signaling, the regulation of adiponectin secretion, inflammation, glucose production, oxidative stress, mitochondrial function, and circadian rhythms [44].

One of the possible causes of obesity relates to the following mechanism: The decreased enzymatic activity of SIRTs, particularly SIRT1, due to excessive glycolytic activity, leads to the accumulation of ROS and the subsequent impairment of the physiological antioxidant mechanism. This, in turn, leads to the development of various metabolic disorders [40]. Conversely, the overexpression of SIRT1 in conditions such as physical activity, calorie restrictions, and fasting leads to the modulation of pro-inflammatory mediators with the suppression of inflammatory signaling pathways and, subsequently, improves glucose tolerance, decreases hyperinsulinemia, and systemically increases sensitivity to insulin.

In the next paragraphs, we summarize the main in vitro and in vivo studies that have investigated the role of SIRTs in obesity.

#### 2.2.1. In Vitro Studies

In vitro studies have shown the important role of SIRT enzymes in the regulation of lipid and glucose metabolism, as well as in the control of adipogenesis.

##### In Vitro Studies: SIRTs and Lipid Metabolism

SIRTs control the biosynthesis of lipids, lipid storage, and lipid utilization, both directly (in the liver, skeletal muscle, WAT, and BAT) and through the activation of insulin secretion. Insulin promotes the storage of fatty acids on WAT and inhibits their oxidation in the liver and skeletal muscle. The role of SIRTs in lipid metabolism is not singular. During stressful events such as fasting, cold exposure, calorie deprivation, and dietary restrictions, SIRT1 and SIRT2 promote lipolysis in WAT, SIRT3 enhances thermogenesis in BAT, and SIRT1 and SIRT7 protect the skeletal muscle and the liver from excessive lipid accumulation [45]. In contrast, SIRT4 and SIRT5 stimulate lipogenesis and inhibit the oxidation of fatty acids.

##### In Vitro Studies: SIRTs and Adipogenesis

SIRT1, by interacting with PPAR, the nuclear receptor co-repressor (N-CoR), the silencing mediator of retinoid, and thyroid hormone receptors (SMRT) attenuates adipogenesis, while SIRT2 suppresses adipocyte differentiation by deacetylating FOXO1 and enhancing FOXO1′s repressive interaction with PPAR-γ [46]. Therefore, the overexpression of SIRT2 inhibits adipogenesis. Some studies have demonstrated that this effect is amplified by the association of a low-calorie dietary regime, leading to new suggestions of therapeutic associations [47]. In contrast, SIRT7 enhances adipocyte differentiation even if, or when, its overexpression does not restore pre-adipocyte differentiation, suggesting that SIRT7 is not enough to activate the program of adipogenesis. Therefore, it can be suggested that the molecules that activate SIRT1 and SIRT2, or inhibit SIRT7, may be a therapeutic strategy for the treatment of obesity [48].

A study conducted on subcutaneous adipose tissue collected from people with a different representation of fat mass (normal weight, overweight, or obese) evaluated the levels of the expression of SIRT genes and their target genes (PPAR-α, PGC1-α, NRF1, DGAT1, PPAR-γ, and FOXO3a). Results showed a different expression of genes encoding SIRTs and their target genes in the adipose tissue of overweight and obese patients when compared with normal-weight controls, suggesting that the adipose tissue modifies its pattern of gene expression [49]. Specifically, the authors described a significantly decreased expression of SIRT1, SIRT3, and SIRT6 mRNA in overweight and obese patients compared to normal-weight subjects. Regarding SIRT1 targets, they found that PPAR-γ, PGC1-α, and FOXO3a mRNA levels were higher in overweight and obese patients compared with normal-weight controls. In contrast, DGAT1, a target gene of SIRT6, showed a lower expression profile in the overweight and obese subjects, compared to normal-weight subjects [49].

##### In Vitro Studies: SIRTs and Glucose Metabolism

The mechanism by which SIRTs interact with glucose metabolism involves glucose homeostasis, insulin secretion, insulin sensitivity, and the regulation of glycolysis and gluconeogenesis. SIRT1 is responsible for glucose homeostasis, the response to oxidative stress, and apoptosis survival. Specifically, the local overexpression of SIRT1 in pancreatic ß-cells results in enhanced insulin secretion, leading to a better response to glucose in vitro and improved glucose tolerance in vivo [50]. The overexpression of SIRT1 also results in an induction of cell survival, preserving cells from damage-induced apoptosis. SIRT1 is also involved in gluconeogenesis control, leading to the decreased synthesis of glucose during fasting [51] and the improvement of insulin sensitivity in muscle cells and adipose tissue through interactions with the tyrosine phosphatase 1B gene, a negative regulator of the insulin signaling pathway (PTP1B), the glucose transporter type 4 (GLUT4), and the nuclear factor ĸb (NFĸB).

In contrast, mitochondrial SIRT4, selectively expressed in pancreatic ß-cells, acts as a negative regulator of insulin secretion by the ADP-ribosylation of glutamate dehydrogenase (GDH), promoting insulin secretion in response to glucose and amino acids when knocked out. Its overexpression, however, has the opposite effect [52,53]. SIRT6 normally competes with the hypoxia-inducible factor 1α (HIF1α), inhibiting the expression of glycolytic genes when glucose intake is adequate. Its deficiency leads to increased HIF1α activity with a consequent increase in glycolysis and a reduction in mitochondrial respiration [54]. Similarly, the absence of SIRT6 in mice enhanced glycolysis and triglyceride synthesis, with the consequent onset of nonalcoholic fatty liver disease (NAFLD) [55].

#### 2.2.2. In Vivo Studies

In vivo studies in experimental animals, in which SIRT genes were silenced or overexpressed, add further evidence that consolidates the role of SIRTs in the pathogenesis of obesity.

In vivo studies on transgenic mice, with the deletion of exon 4 in the SIRT1 gene, showed an increased rate of hepatic steatosis when fed with a high-fat diet [56]. Wang and colleagues, in their study on mutant mice with the deletion of exons 5 and 6 in the SIRT1 gene, reported an increased frequency of fatty liver disease, lipid deposition, and higher triglycerides in plasma and the liver, thus providing further evidence that an SIRT1 deficiency causes fatty liver diseases even without a high-fat diet. SIRT1 knock-out mice showed hyperglycemia and insulin resistance due to the increased hepatic gluconeogenesis and the increased intracellular ROS accumulation in multiple tissues, including the liver, adipose tissue, skeletal muscle, and spleen [57]. Thus, the selective interruption of SIRT1 signaling in the liver not only causes hepatic steatosis but also promotes the progression of advanced metabolic syndromes, asserting the role of SIRTs in systemic inflammation. Conversely, mice with an SIRT1 overexpression have increased lipolysis and decreased oxidative stress and inflammation that leads to a lower liver lipid overload, reduced liver injury, and reduced fibrosis [58].

Mariani and colleagues showed the correlation between SIRT levels in plasma and the different conditions related to adiposity, fat content, fat distribution, energy homeostasis, and inflammation. The population studied included under-weight, normal-weight, and obese subjects [59]. The parameters evaluated were epicardial fat thickening (an important predictor of adipose visceral mass), a metabolic panel (glucose, insulin, LDL- and HDL-cholesterol, and triglycerides), an inflammatory panel (erythrocyte sedimentation rate, C-reactive protein, and fibrinogen), and SIRT1 after 12 h of fasting. The authors found a significant negative correlation between the plasma concentrations of SIRT1 and adipose tissue, as higher levels were present in people with the lowest component of fat mass. Elevated SIRT1 concentrations were also linked to low levels of insulin, LDL cholesterol, and insulin resistance. These patients also had a lower pro-inflammatory state, confirming the assumption that obesity, a condition associated with low circulating levels of SIRTs, is a disease where inflammation plays an important role [59].

### 2.3. Sirtuins and the Male Reproductive System

**Figure 1:**
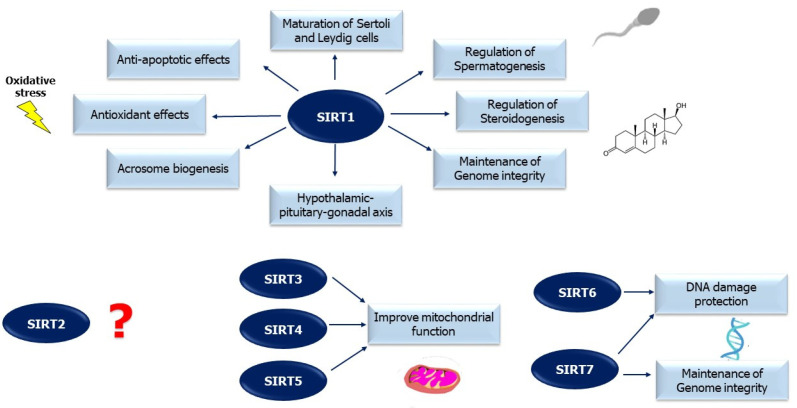
Sirtuins (SIRT) and male reproduction. This figure summarizes the main effects of SIRTs on male reproductive function. SIRT1, the most studied member of the SIRT family, seems to have a pivotal role in male fertility through several mechanisms. Conversely, the role of SIRT2 in male fertility is completely unknown. There is little data on the relationship between other sirtuins and male reproduction.

The role of SIRTs in male fertility is not well known (Figure 1). Only a few studies have evaluated this relationship, showing the possible role of SIRTs on male reproductive function. Indeed, SIRTs are highly expressed in mammalian testicular tissue and appear to play an important role in maintaining spermatogenesis [37].

#### 2.3.1. SIRT1

SIRT1 is one of the most studied members of the SIRT family [34]. SIRT1 knock-out mice (Sirt1-/-) have small and abnormal seminiferous tubules and a reduced number of spermatozoa in the testes and epididymis [60]. Therefore, SIRT1 deficiency alters spermatogenesis, and this study suggests its pivotal role in this process. Another study showed that Sirt1-/- mice have an almost complete absence of the tubular lumen [61]. Given that the appearance of the lumen in the seminiferous tubules is a marker of Sertoli cell differentiation, these results suggest that SIRT1 is involved in the maturation of Sertoli cells. This hypothesis is further supported by the reduced expression of reproductive homeobox 5 (RHOX5), an androgen-regulated homeobox gene specifically expressed in these cells. Leydigian function also appears to be impaired in SIRT1-/- mice. Indeed, they have a reduced number of adult-type Leydig cells, as well as a down-regulation of genes involved in steroid metabolism, such as the steroidogenic acute regulatory protein (STAR) and cytochrome P450, and lower intratesticular testosterone levels compared to controls. Therefore, these results suggest that the inactivation of SIRT1 does not affect the development of fetal Leydig cells, but their maturation. Low levels of LH and FSH have also been described in SIRT1-deficient mice [61]. This suggests that SIRT1 also acts at the hypothalamic-pituitary level to alter gonadal function.

Meiosis and DNA replication are two fundamental aspects of spermatogenesis. Kolthur-Seetharam and colleagues reported that spermatogenesis arrests during the late meiotic prophase in SIRT1-/- mice. This conclusion comes from the presence of many multinucleate, or abnormally large, spermatocytes in the seminiferous tubules of mutant mice, associated with the down-regulated expression of late-meiotic and post-meiotic genes, whereas the pre-meiotic genes were not altered. SIRT1 may also play a role in maintaining genomic integrity. Indeed, SIRT1-deficient mice showed a significantly elevated number of spermatozoa with single or double-strand DNA breaks [61].

SIRT1 also protects against apoptosis. SIRT1-/- mice showed an excessive number of apoptotic cells in the seminiferous tubules [62]. Increased oxidative stress is a well-known cause of sperm DNA damage. Varicocele increases oxidative stress that, in turn, results in male infertility. Mostefa and colleagues reported an SIRT1 deficiency, and increased oxidative stress, as causes of male infertility in patients with varicocele [63]. Indeed, SIRT1 has antioxidant effects [64], whereas varicocele leads to oxidative stress. Thus, the presence of varicocele associated with a SIRT1 deficiency amplifies DNA damage and causes infertility. A case-controlled study reported a lower seminal expression of SIRT1 in patients with oligoasthenoteratozoospermia (especially in those with varicocele), compared to fertile men [63]. The same group of researchers subsequently showed increased levels of SIRT1 after varicocele surgical repairs and, interestingly, these levels correlated positively with sperm concentrations, motility, and normal morphology [65].

Globozoospermia is a rare cause of male infertility. This condition is characterized by the presence of round-headed spermatozoa. The morphological abnormality is due to the absence of an acrosome, or the presence of an abnormal acrosome. SIRT1 appears to be involved in the formation of the acrosome cap. Indeed, Liu and colleagues showed that a SIRT1 deficiency in germ cells leads to an accumulation of acetylated LC3, the light chain of the autophagic molecular marker microtubule-associated protein, in the germ cell nucleus. Since SIRT1 is involved in the deacetylation of LC3, which is essential for the redistribution of LC3 in the cytoplasm, it could play a role in the biogenesis of acrosome during spermiohistogenesis by modulating autophagy [66].

#### 2.3.2. SIRT2

The role of SIRT2 in male infertility is completely unknown. However, an interesting relationship between SIRT2 and microtubules has been proposed [34]. Microtubules play a fundamental role in cellular shape, transport, motility, and cell division [34]. They are formed by α- and β-tubulin heterodimers. In vitro and in vivo studies reported the deacetylase activity of SIRT2 on α-tubulin [67]. Specifically, SIRT2 deacetylates Lys40 in α-tubulin. The histone deacetylase HDAC6, a class II HDAC, has a similar activity and deacetylates the same residue of α-tubulin [67]. It was previously shown that HDAC6 inhibition led to an increase in the acetylation of α-tubulin that, in turn, altered sperm motility [68]. SIRT2 and HDAC6 are found along microtubules and both proteins co-immunoprecipitate after their co-expression in cells [67]. Therefore, these results suggest that both SIRT2 and HDAC6 are part of a multi-protein complex that can regulate the level of tubulin acetylation [67]. Assuming that both SIRT2 and HDAC6 deacetylate α-tubulin, it can be hypothesized that SIRT2, also by deacetylating α-tubulin, has an important function on sperm motility and, therefore, on male reproduction.

#### 2.3.3. SIRT3

SIRT3 is one of the three mitochondrial sirtuins, where the others are SIRT4 and SIRT5. It is well-known that mitochondria have a fundamental role in the production of ATP and that mitochondrial dysfunctions contribute to the alteration of the redox balance, predisposing aging and metabolic alterations [69]. Several studies have shown the role of SIRT3 in controlling ATP levels and its antioxidant properties. The absence of SIRT3 deacetylase activity causes a significant reduction in ATP levels in various tissues, especially in the liver, heart, and kidney. It would be interesting to understand if the lack of SIRT3, and the consequent reduction in ATP levels, could lead to important metabolic alterations in some cells and tissues in various stress conditions [70], such as in male germ cells during the fine process of spermatogenesis. A case-control study showed lower levels of SIRT1 and SIRT3 in the seminal plasma of patients with asthenoteratozoospermia, compared to normozoospermic men [71].

SIRT3 plays an important role in the caloric restriction-mediated prevention of oxidative damage by improving the mitochondrial glutathione antioxidant defense system [72]. SIRT3 may have a central role in aging retardation in mammals. Considering the role of aging and obesity in male infertility, it would be interesting to evaluate the effects of caloric restriction on SIRT3 antioxidant activity in male germ cells and, hence, on male reproductive function. After all, SIRT3 protects ovaries, oocytes, and early embryos against stress conditions, pointing out its role in female fertility [69].

#### 2.3.4. SIRT4

Little is known about SIRT4. It seems to possess a peculiar ADP-ribosyl transferase activity in addition to the deacetylase activity that distinguishes this class of proteins. Furthermore, SIRT4 plays a role in energy homeostasis, ATP production, and glutamine metabolism, thus becoming a major factor in mitochondrial dynamics and redox homeostasis [69]. The bacterial lipopolysaccharide (LPS) is one of the most important factors in the pathogenesis of bacterial infections in the male accessory glands and can inhibit testicular steroidogenesis and induce apoptosis. LPS has been reported to cause mitochondrial dysfunction by suppressing SIRT4 which, in turn, affects Leydig cell functions by modulating steroidogenesis and apoptosis [73].

#### 2.3.5. SIRT5

SIRT5 exerts several functions on mitochondria. Indeed, a lack of SIRT5 is associated with the highest degree of mitochondrial fragmentation [69]. The mitochondrial homeostatic activity of SIRT5 may play a role in male fertility, considering the large number of mitochondria present in the microtubules of the sperm tail and their important role in sperm motility [74]. Furthermore, it is known that mitochondria play a role in germ cell proliferation, mitotic regulation, and the elimination of germ cells by apoptosis [75].

Bello and colleagues reported a reduction of the expression of mitochondrial sirtuins, evaluated by q-RT PCR, in the semen of infertile patients compared to fertile men. The authors also observed a positive correlation of the expression of the heat shock protein 90 (HSP90) with SIRT3, SIRT4, and SIRT5 expressions in the semen of fertile men, whereas a negative correlation was observed between HSP90 in the semen of infertile men and mitochondrial sirtuin genes in the semen of fertile men. These findings suggest that the dysregulation of mitochondrial sirtuin genes causes mitochondrial dysfunction due to stress, which, in turn, appears to be associated with human male infertility [76].

#### 2.3.6. SIRT6

A very small number of studies have investigated the role of SIRT6 in male fertility. Mice fed a high-fat diet for 16 weeks showed a significant decrease in SIRT6 levels in the nucleus of transitional spermatids and in the acrosome of mature spermatozoa [77]. These findings were associated with high levels of acetylated H3K9 in the nucleus, as well as increased DNA damage, suggesting a possible role of SIRT6 in spermatogenesis [77]. Another study confirmed that SIRT6 protects cells from DNA damage [78]. The authors found that SIRT6 stimulates double-strand break repair pathways in mammalian cells exposed to increased levels of oxidative stress. Although this study was not conducted on germ cells, it cannot be ruled out that this sirtuin exerts similar effects on these cells and, consequently, on male reproductive function.

#### 2.3.7. SIRT7

SIRT7 is functionally linked to transcriptional regulation by controlling ribosome production that directly acts on RNA polymerase I (Pol I) [79]. Indeed, the overexpression of SIRT7 increases Pol I-mediated transcription, whereas the knockdown of the SIRT7 gene, or the inhibition of its catalytic activity, results in the decreased association of Pol I with rDNA and a reduction in Pol I transcription [80]. Vazquez and colleagues showed the role of SIRT7 in the maintenance of genome integrity. Indeed, SIRT7 knockout mice had a reduced embryonic viability and those who survived had a progeroid-like phenotype. The authors also found replication stress and an impaired DNA damage response in SIRT7-deficient cells [79]. Therefore, the role of SIRT7 in genome integrity and embryonic viability may suggest a significant impact on reproduction.

## 3. Sirtuins: Possible Molecular Targets for the Treatment of Obesity-Induced Male Infertility

There are strategies that attempt to improve the activity of SIRTs to counteract the harmful effects of metabolic disorders [81]. In parallel, these approaches could also be used to counteract infertility caused by excess weight in infertile male patients. In this regard, several molecules have been studied as activators of SIRTs and, particularly, of SIRT1 [82]. These include resveratrol (RSV), metformin, berberine, quercetin, SRT1460, SRT1720, and SRT2183.

RSV, also known as 3,4′,5 trihydroxystilbene, is a polyphenolic compound found in grapes, peanuts, berries, and red wine [83,84]. It is synthesized from the aromatic amino acid phenylalanine through the activation of the enzyme stilbene synthase, and it exists in two isomeric forms, trans- and cis-resveratrol [85], where the former is the most common. In 1997, the anticancer properties of RSV were first described [86]. Since then, several other properties were attributed to this substance, including antioxidant and anti-inflammatory effects, as well as anti-lipid, anti-aging, anti-bacterial, anti-viral, and neuroprotective actions [87]. Moreover, the administration of RSV seems to have a beneficial role in obesity by reducing body weight, BMI, waist circumference, and fat mass [88], as well as improving glucose homeostasis and attenuating the cardiovascular risk associated with obesity [87]. RSV regulates energy metabolism and mitochondrial activity, thus protecting against metabolic diseases [89]. It stimulates SIRT1 deacetylase, which, in turn, inhibits adipogenesis and adipocyte differentiation [87]. The inhibition of adipogenesis may be mediated by SIRT1 directly or indirectly by increasing the expression of the Forkhead box protein O1 (FoxO1) [90]. SIRT1 also activates PGL-1α and, consequently, stimulates mitochondrial activity and glycogenesis, as well as inhibiting glycolysis in the liver [89]. RSV also increases the activity of the adenosine monophosphate-activated protein kinase (AMPK) [91], which hinders adipogenesis.

On this basis, it has been hypothesized that RSV may be effective for the treatment of infertility in obese males. Experimental studies described a protective role of RSV in the sperm function of obese animals [92,93,94]. In high-fat-fed mice, RSV was reported to improve testosterone levels by upregulating the StAR enzyme [92].

**Figure 2:**
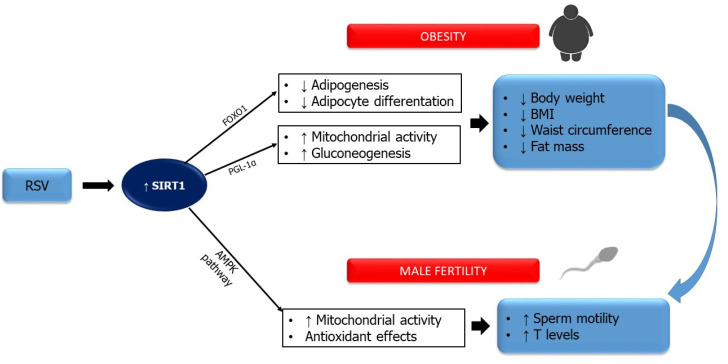
Effects of sirtuin-1 (SIRT1) activation by resveratrol (RSV) on obesity and male fertility. Strategies that attempt to improve SIRT activity could be used to counter infertility caused by excess weight in infertile male patients. Several molecules have been studied as activators of SIRTs, particularly RSV. Indeed, its administration seems to have a beneficial role both in obesity by reducing body weight, body mass index, waist circumference, and fat mass, and on fertility by increasing testosterone levels. Abbreviations: AMPK: monophosphate-activated protein kinase; FoxO1: Forkhead box protein O1; PGC-1α: peroxisome proliferator-activated receptor γ coactivator-1α; SIRT-1: deacetylating enzyme sirtuin-1.

To the best of our knowledge, only one study has investigated the effects of RSV on sperm parameters in obese men [95]. Cui and colleagues [95] evaluated the effects of RSV treatment on sperm motility, plasma zinc concentrations, and acrosin activity in the semen of obese patients. Semen samples from obese and asthenozoospermic patients, incubated with increasing concentrations of RSV, showed varying degrees of improvement in sperm motility [95]. They also described a significant increase in seminal plasma zinc concentrations and sperm acrosin activity, compared with the control group. These results suggest that RSV could have a therapeutic and protective effect against obesity-induced alterations of the sperm parameters (Figure 2).

SIRT1 also appears to mediate the beneficial effects of metformin [82]. Metformin is an insulin sensitizer drug belonging to the biguanide class of oral hypoglycemic agents and it is the first-line drug for the treatment of type 2 DM. It increases the tissue sensitivity to insulin, prevents inflammation and oxidative stress in the tissues, and exerts a weight-loss favoring effect [96]. Previous studies reported a positive effect of metformin on the male reproductive alterations induced by metabolic syndromes [97,98]. Metformin improves male function by different mechanisms. AMPK appears to be the main target of metformin in the testes and, in turn, the normalization of the AMPK-dependent pathways in the testes, by metformin, may be the major mechanism of its efficacy in improving male reproduction in patients with metabolic diseases [96]. Indeed, AMPK regulates the growth and differentiation of Sertoli and Leydig cells, as well as controlling sperm motility and acrosome reactions [99]. Metformin is also used in women with polycystic ovarian syndrome (PCOS) due to its insulin sensitizer activity [100]. Interestingly, in PCOS rats, the SIRT1 expression is significantly up-regulated after the administration of metformin [101]. Moreover, metformin increases the SIRT3 expression and activity in oocytes of PCOS mice [102]. At the testicular level, metformin rescues male reproductive alterations induced by type 2 DM in rats fed a high-fat diet, as well as up-regulating the expression of SIRTs alone or in combination with other substances [103,104].

Other natural compounds appear to modulate SIRT activities and they have been studied for the treatment of women with PCOS. These include quercitin and berberine [69]. Quercetin, the most abundant dietary flavonoid, counteracts the altered levels of adiponectin, visfatin, and resistin in PCOS women and up-regulates the expression of AMPK and SIRTs in ovarian tissue [105]. Berberine, a yellow alkaloid naturally produced by several plants, improves insulin sensitivity and ovulation function in PCOS patients by promoting the cell glucose uptake via SIRT3 ubiquitination, as well as activating the AMPK pathway [106]. Furthermore, several other small molecules (SRT1460, SRT1720, and SRT2183) appear to activate SIRT1. These compounds were found to be approximately 1000-fold more potent than resveratrol. Among them, SRT1720 appears to be the most promising SIRT1 activator. Its administration improved glucose homeostasis, increased the sensitivity to insulin, and improved mitochondrial function in type 2 DM mouse models [107].

Finally, diet and physical activity play a main role in counteracting the negative effects of obesity on male reproduction. In recent decades, the use of a very low-calorie ketogenic diet (VLCKD) has gained growing interest in the treatment of obesity and its comorbidities. This therapeutic dietary intervention mimics fasting by markedly lowing carbohydrates intake (<30 g/day), thus increasing ketone biosynthesis. Ketones are used as fuel by many tissues, including the central nervous system, skeletal muscle, and the heart [108]. Very recently, VLCKD has been shown to be efficacious in the treatment of metabolic hypogonadism in overweight/obese patients [31,108,109]. Therefore, it seems that VLCKD could represent an effective strategy against obesity and its reproductive and sexual complications. SIRT1 is characteristically upregulated by fasting. The stimulation of SIRT1 promotes ketogenesis, gluconeogenesis, and fatty acid oxidation via two downstream effectors, PGC-1α, and the fibroblast growth factor 21 [110]. However, few studies have investigated the relationship between SIRT1 and ketogenesis and their results showed a complex and unpredictable relation [110]. Further studies investigating the relationship between the ketogenic diet and SIRT1 are needed.

Overall, further studies are needed to explore the effects of natural and synthetic substances and lifestyle changes on SIRT activity and, in turn, the potential benefits that SIRT activation can have on obesity-related male infertility.

## 4. Conclusions

In recent decades, a progressive decline in sperm quality and male fertility has been described [8]. At the same time, lifestyle factors including erroneous eating habits and sedentary lifestyles have dramatically increased the prevalence of obesity in Western countries. Several studies have reported the close relationship between obesity and male reproductive dysfunction. Among the mechanisms by which obesity impairs male reproductive function, SIRTs have an emerging role.

SIRTs regulate energy balance, lipid and glucose metabolism, and adipogenesis; likewise, current evidence also confirms the role of SIRTs in male reproduction. However, the majority of the studies have been conducted on animal models and very few have been conducted in humans. This review shows that SIRTs have an important role in obesity-related male infertility, and it underlines the need to further investigate this relationship. It will be particularly interesting to understand whether synthetic or natural compounds capable of modifying SIRT activity may be useful for the treatment of obesity and its associated complications, including obesity-induced male infertility. The relationship between SIRTs, female fertility, and obesity has been more investigated, especially in PCOS patients and in experimental models. However, as we have shown, very few studies clarify the role of SIRT activators in obesity-related male infertility. These studies suggest the role of RSV in modulating SIRT activity to counteract the negative effects of obesity on male fertility. However, to the best of our knowledge, only one study has been conducted in humans and it shows the beneficial effect of RSV treatment on sperm parameters in obese patients [95]. Further studies are needed to evaluate the efficacy of this flavonoid in obese patients with altered sperm parameters.

The search for strategies to improve male reproductive function in overweight/obese patients is a challenge and understanding the role of SIRTs and their activators may open new interesting scenarios in the coming years.
